# Reputation and trust in health insurance: A scoping review of key drivers and outcomes

**DOI:** 10.1371/journal.pone.0345875

**Published:** 2026-03-27

**Authors:** Gaël Saillen, Nicola Diviani, Sara Rubinelli

**Affiliations:** 1 Faculty of Health Sciences and Medicine, University of Lucerne, Lucerne, Switzerland; 2 Swiss Paraplegic Research, Nottwil, Switzerland; Yeungnam University College of Pharmacy, KOREA, REPUBLIC OF

## Abstract

**Background:**

Reputation is a critical factor in the health insurance industry, impacting business performance and public health outcomes. However, despite its acknowledged importance, the reputation of health insurers has received limited attention in both academic research and industry practice. Gaining a comprehensive understanding of health insurers’ reputation could help insurers strategically manage their public image while providing policymakers with valuable insights to develop policies that improve health outcomes.

**Objective:**

This study aims to identify the factors shaping the reputation of health insurers and to assess the broader impacts of reputation within the industry and the health care system.

**Design:**

A scoping review was conducted to map the existing literature on the reputation of health insurers. Using the PRISMA-ScR framework, it identified key determinants and outcomes of health insurer reputation, clarified concepts of reputation and trust, and highlighted gaps in the current body of literature.

**Results:**

A total of 3385 records were identified, of which 397 were fully screened for eligibility, resulting in the inclusion of 45 studies. The majority of the included studies conducted surveys (n = 40), while the minority employed qualitative interviews (n = 6) and focus group discussions (n = 7). The total number of participants involved in the included studies is 31’344. The analysis identified 23 descriptives themes, which were organised into two main analytical categories: determinants and outcomes. The determinants included 17 descriptive themes which have been further classified into three groups based on whether they pertain to policyholders, insurers or providers. The outcomes consist of six key descriptive themes.

**Conclusion:**

This review provides a thorough analysis of research that have investigated different aspects of health insurance reputation and identify significant gaps in the literature. It maps key determinants of reputation – such as corporate culture, product structure, and service quality – that insurers can directly influence. Additionally, factors like the quality of healthcare providers and targeted communication efforts may also be linked to reputation. The analysis underscores the importance of reputation for core business activities (e.g., client acquisition and retention) and its role in preventive healthcare. Notably, there is a lack of research on the regulatory implications of insurers’ reputation, suggesting a need for further studies to enhance preventive strategies and integrate insurers into a broader public health framework.

## 1. Introduction

Reputation plays a critical role in the health insurance industry, where the intersection of healthcare financing, cost considerations, and regulatory frameworks influences operational outcomes and stakeholder trust. While reputation is widely recognized as a key factor in business success, its specific relevance to health insurers remains underexplored, despite growing attention in management research, as highlighted by Veh [1 p. 316] and Chun [2 p. 94]. This review addresses this gap by systematically analyzing the determinants and outcomes of health insurers’ reputation, moving beyond financial metrics such as premium costs to explore its broader implications. Recognizing reputation and trust are distinct yet related concepts, we considered both in the conceptual framing of our research. While we privilege the term reputation, we refer to trust when it is the primary focus in the studies cited.

The importance of trust in health insurance systems was articulated by Arrow, who argued that trust is a necessary condition for the functioning of markets characterized by uncertainty and information asymmetry [3 pp. 965−366]. In this contexte, the financial consequences of health risks are delegated to third parties, placing individuals in a relationship of dependence that relies on trust. Building on this foundational insight, subsequent scholarship has expanded and refined Arrow’s argument. Hall [4] emphasizes that trust in healthcare and insurance contexts is not only a response to statistical or financial uncertainty, but also to the psychological vulnerability associated with illness and treatment. From this perspective, trust may emerge as a relational construct shaped by experiences of dependence, exposure, and perceived institutional integrity, thereby closely aligning with the notion of organizational reputation. Complementing these perspectives, institutional approaches such as Scharpf’s actor-centered institutionalism [5] highlight that reputation and trust are embedded within institutional contexts that structure actors’ incentives, constraints, and strategic interactions. In regulated insurance systems, reputation thus functions not only as an attitudinal outcome, but also as a strategic resource shaped by and responsive to institutional rules and policy environments.

More recent work in behavioral health economics shows that health insurance choices are strongly shaped by behavioral frictions, including information search costs, choice complexity, inertia, and limited awareness of plan attributes [6–8]. In this contexte, such frictions may increase the saliance of insurer reputation in shaping the relationship between policyholders and insurers. They also raised reflection about the adequacy of existing legislative and regulatory frameworks.

From a public health perspective, the reputation of health insurers extends beyond a business asset to a potential public health priority. A positive reputation can enhance customer retention, foster investor confidence, and improve employee satisfaction (1, p. 323). Moreover, it can strengthen collaboration with public health systems, promote compliance with preventive health measures, and support a culture of continuous improvement in healthcare practices [9]. Conversely, a poor reputation may increase regulatory scrutiny and undermine efforts to develop and implement effective health policies. These dynamics underscore the dual importance of reputation in shaping both business success and public health outcomes.

This scoping review aims to provide the first comprehensive synthesis of existing literature on health insurer reputation. Specifically, it seeks to identify the key factors shaping reputation and assess its impact on insurers, policyholders, and the broader healthcare system. By clarifying these determinants and exploring their consequences, the review aims to equip insurers with actionable insights for strategic reputation management and inform policymakers about how reputation can be leveraged to enhance health outcomes. Additionally, the study highlights critical gaps in the current literature, paving the way for future research to build a deeper understanding of this essential topic.

## 2. Method

To achieve the study’s objective, we conducted a scoping review, an approach ideally suited for mapping the extent and breadth of existing literature on a given topic – in this case, the reputation of health insurers [10]. Scoping reviews enables a comprehensive mapping of key themes and knowledge gaps, with a focus on conceptual and contextual dimensions. The review adhered to the PRISMA guidelines for scoping reviews (PRISMA-ScR) [11], ensuring a rigorous and structured methodology. By systematically exploring the diverse body of literature on health insurer reputation, this approach clarifies key concepts such as reputation and trust within the health insurance context and support the identification of critical gaps, therby providing a foundation for future research.

### 2.1. Search strategy

We conducted a systematic search of peer-reviewed literature on the reputation of health insurers using PubMed, Scopus, and Web of Science.

Our search strategy was organized around two primary conceptual blocks. The first block focused on terms related to “health insurance,” which was relatively straightforward to define. The second block encompassed terms associated with “reputation,” which required a more nuanced approach. To guide this process, we referred to Fombrun’s widely accepted definition: “A corporate reputation is a perceptual representation of a company’s past actions and future prospects that describes the firm’s overall appeal to all of its key stakeholders when compared with other leading rivals” [12]. This definition highlights that reputation reflects the aggregation of stakeholder perceptions. Recognizing the diversity in how reputation is defined, we also included related terms such as “corporate identity” and “perceived quality” to capture the broader conceptual scope.

Additionally, we accounted for the distinction between reputation and trust, which is not always clear-cut. Veh identifies “reputation as an antecedent of trust” (1, p. 323), while trust itself has been described as the willingness of one party to be vulnerable to the actions of another. Hall further distinguishes between “social trust” and “interpersonal trust” [13]. In light of these nuances, we included “trust” as part of our search terms to ensure comprehensive coverage.

The final search strategy, conducted on 9 January 2026, incorporated the following keywords: “health insur*”; “health plan*”, “managed care”, reputation; “corporate image”; “corporate identity”; “reputational status”; ethos; fame; esteem; “perceived quality”; “Prominence”; and trust. Detailed information on the database search blocks is provided in S1 Appendix.

### 2.2. Inclusion and exclusion criteria

Eligibility criteria were applied. We included papers written in English, only original studies and peer-reviewed articles. To maintain conceptual specificity and manageability, we restricted inclusion to studies containing “insur*”, “health plan*” or “managed care” in the title to ensure that insurance organisations or systems constituted the primary object of analysis. In contrast, reputation- and trust-related concepts were searched more broadly, reflecting their heterogeneous use across disciplines. This asymmetric strategy balanced precision and recall and supported a focused and analytically coherent mapping of the literature on health insurer reputation To focus on high-quality empirical evidence, we excluded grey literature, editorials, dissertation and opinions. We have excluded the records published before 1990, as it was not until the 1990s that studies on reputation really emerged (1 p. 316).

### 2.3. Selection of studies and data extraction

For the purpose of the current review, we extracted descriptive data about the study (author(s), title, year, country/countries of data collection, study design, method of data analysis), on the population (policyholders, household, student, sample size), the concept used (reputation or trust, industry of insurers), the purpose (research objective or question and determinant, outcome or assessment), and the main findings.

Coding was performed collaboratively by the research team. An initial coding draft of the qualitative and quantitative results related to the outcomes discussed in the present review was produced by the first author. To maintain uniformity in the coding and interpretation of data, the author team met on multiple occasions to discuss the process, refine and modify the coding. The authors reached a consensus on the coding technique and rules to be implemented for the remaining articles after deliberation.

### 2.4. Synthesis of results

We grouped the studies based on the specific area of their analysis. The first category includes all studies that addressed the determinants of the reputation of health insurers. This also includes the majority of studies that focus on assessing the reputation of insurance companies, as the items used are often determinants. A second group focused on outcomes. Detailed subgroup analyses were conducted on the World Bank classification of Gross National Income [14] (high-, upper-middle-, lower-middle-, or low-income countries) and health expenditure according to the WHO [15] (government schemes, compulsory insurance, voluntary insurance, household out-of-pocket), see S3 Appendix for full search strategy. The significance of this feature lies in its influence on the insurers’ reputation. Their importance in financing depends on the share of financing they represent, and the possible obligation to be insured. We summarized the results in tabular format.

## 3. Results

The total number of entries listed after the search is 3385 (1837 on Scopus, 493 on PubMed and 1055 on Web of Science). The final search results were exported to EndNote, where 1000 duplicates and 83 ineligible records (70 prior to 1990) were automatically excluded. 270 duplicates were additionally manually removed.

**Fig 1:**
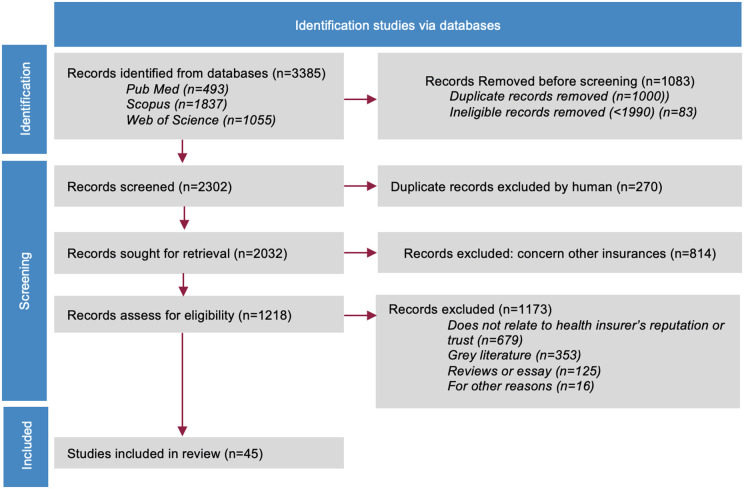
Overview of the selection process. (PRISMA-ScR).

The remaining 2032 records were assessed for eligibility, of which 1987 entries were excluded. The primary reason for exclusion was that the records focused on other types of insurance, accounting for 814 records. Additionally, 679 were excluded due to a lack of relevance to reputation or trust in health insurance, 353 because they were grey literature or not peer-reviewed, 125 because they were essays, and 16 for other reasons. In total, 45 studies were included in our analysis, as shown in **Fig 1**.

The search on the prementioned databases was completed by a search on Google Scholar. No relevant additional record was found. PRIMSA for scoping reviews (PRISMA-ScR) checklist was used for the reporting of findings.

### 3.1. Characteristics of included studies

The main characteristics of the included studies are summarized in Table 1 to provide a high-level overview of the evidence base. Detailed study-level information is presented in S2 Appendix.

**Table 1:** Overview of characteristics of included studies.

Characteristic	Summary
Total number of studies	45
Geographical distribution	Africa (n = 12); Asia (n = 17); Europe (n = 11); North America (n = 5); Oceania (n = 1)
Study design	Surveys (n = 40); Qualitative interviews (n = 6); Focus groups (n = 7)*
Survey mode	Online (n = 16); Telephone (n = 6); Postal (n = 5); Face-to-face (n = 5); Not specified (n = 8)
Sample size (quantitative studies)	Range: 150–7097 participants
Sample size (qualitative studies)	Range: 10–353 participants
Total participants across studies	31’344
Study population	Policyholders only (n = 23); Insured and uninsured (n = 21); Other (general practitioners, school setting, female traders) (n = 3)
Primary unit of analysis	Insurer as organization (n = 40); Insurance sector/system (n = 8)
Primary concept studied	Trust (n = 40); Reputation (n = 7); Other related concepts† (n = 11)

† *Most frequently: perception (n = 4).*

* *Some studies used multiple qualitative methods.*

### 3.2. Data synthesis

Our analysis of the included studies identified 23 descriptive themes, which were further synthesized into two overarching analytical themes: “Determinants” and “Outcomes”. These analytical themes provide a structured framework to understand both the factors influencing health insurers’ reputation and the consequences of that reputation. Two studies focusing exclusively on the assessment of reputation were not included in this thematic synthesis [16,17].

“Determinants” refer to the underlying factors or characteristics that shape or influence the reputation of health insurers. These factors may arise from the perspectives of key stakeholders, including policyholders, insurers themselves, and healthcare providers. They encompass elements such as customer satisfaction, perceived service quality, corporate culture, communication strategies, and trust in healthcare providers. “Outcomes” reflect the effects or consequences of health insurers’ reputation on their operations, stakeholders, and the broader healthcare system. These include customer behaviors such as policyholder retention, willingness to pay, and enrollment, as well as aspects related to public trust, data sharing, and collaboration within healthcare systems.

To support this analysis, we included direct extracts from the studies that illustrate key findings. These extracts, systematically linked to their references, provide evidence-based examples of each descriptive theme.

**Fig 2:**
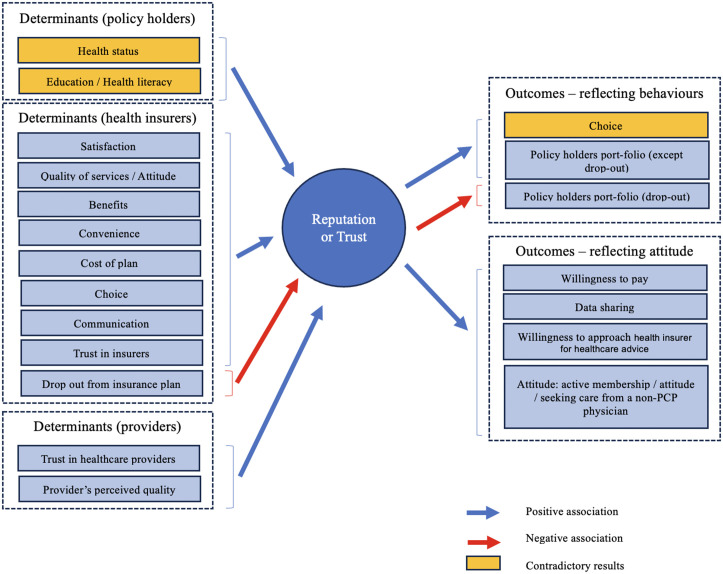
Conceptual framework of determinants and outcomes of health insurer reputation.

For a consolidated view, Table 2 presents a detailed summary of the descriptive themes, categorizing them into determinants and outcomes. The table also includes selected extracts to exemplify how these themes are operationalized within the studies, offering insights into their practical implications and interconnections. In addition, Fig 2 presents a conceptual model illustrating how the identified determinants influence trust and reputation, and how these, in turn, are associated with behavioural and system-level outcomes.

**Table 2:** Overview of descriptive and analytical themes.

Analytical theme	Descriptive theme	Association	Extract	References
Determinants (Policy holders)	Demographic (age, gender, family status, income level, ethnicity)	No association	“We did not find race and trust in insurers to be associated” Goold (2015, p. 71), “The hypothesised interaction effects between self-reported health and trust and between gender and trust were not significant, therefore H2 en H4 are rejected.” Bes (2013, p. 6)	[18,19,20–24]
	Locus of control	No association	“In the fourth block, locus of control was entered. The model was not significant (F =.070, p =.932)” Gabay (2015, ‘. 87)	[19]
	Health status (including visit frequency)	Positive	“Those with fair or poor health status had lower levels of trust in their insurer than those with good, very good, or excellent health status.” S. D. Goold (2015, p. 69)	[18,19,21,22,23,24]
		No Association	“The hypothesised interaction effects between self-reported health and trust and between gender and trust were not significant, therefore H2 en H4 are rejected.” Bes (2013, p. 6)	[24]
	Education/ Health literacy (higher level = higher trust, except for one)	Positive	“The finding reveals that health insurance literacy positively influences perception and affect people’s preference to invest in PVHI.” T. Mathur; “Among demographics only education was significant” G. Gabay (2015, p. 85)	[23] [18,19,25–30]
		Negative	“Respondents with at least a high school degree had lower levels of trust than the less educated. “S. D. Goold (2015, p. 69)	[20]
Determinants (Insurer)	Selection of the health insurer/ type of health insurance	No association	“In contrast to previous studies, the selection of the health-care insurer was not significant and may be explained by the fact that in most cases physicians work with multiple health plans.” Gabay (2015, p.87)	[18,19]
	Satisfaction	Positive	“This study showed that members’ satisfaction with what they experienced at health facilities and local CBHI offices had a positive association with trust in the CBHI scheme.” Eseta (2022, p. 11)	[19,25,27,31,32,33]
	(Perceived) Quality of services/ Attitude	Positive	“Lastly, we identified significant effects of functional quality on trust in the company (p < 0.001) and of technical quality on trust in the company” Wendel (2011, p. 7)	[34,18,19,24–26,31,32, 33,35,–38]
	Benefits	Positive	“The second extracted factor accounted for 15% of variance and was indicative of “benefits of PVHI plans” with the statements: saves money during hospital bills, reduce tax liability, cut dependence on loan and provide long-term financial security against adverse health outcome” Mathur (2018, p.6)	[19] [26]
	Convenience	Positive	“The fifth factor “convenience” accounted for about 5% of variance that dealt with perceptions about locating insurers’ office or distributor, selecting insurance provider, choosing plan and enrollment” Mathur (2018, p.6)	[26]
	Cost of plan	Positive	“The sixth factor “cost of plan” approximately accounted for over a 4% variance in data that exclusively dealt with respondents’ impression about the health plans charges.”	[26]
	Choice (product, etc,)	Positive	“Higher insurer trust was significantly associated with increasing years of enrolment with the insurer and higher adequate choice in insurer selection. “ » Balkrishnan *et al.* (2003)	[18,22]
	Communication (publicity/ perceived communication, culture of transparency, Empathy)	Positive	“This shows that when the company’s publicity is higher, the trust increases as well.” Seiferth (2015, p. 3714); “Customers expected Omega to show empathy by being compassionate, confident, and transparent with them” “. Grundstrom *et al.* (2020, p.8)	[18,21,24,31,33,35,38]
	Trust in insurers	Positive	“The fourth factor “trust” accounted for 7% of variance and captured the extent of respondents’ faith in plans and insurance providers.” Mathur *et al.* (2018, p. 6)	[26]
	Dropout from insurance plan	Negative	“Finally, a dropout from the CBHI scheme has a negative association with trust in the CBHI scheme” Eseta (2022, p. 10)	[25]
Determinants (Provider)	Provider’s communication	No association	“Perceived communication style was not significant.” Gabay (2015, p. 87)	[19]
	Trust in healthcare providers (Change healthcare providers)	Positive	“The most significant predictor of these changes was changing primary care physicians since baseline (OR: 2.17, 95 percent CI: 1.37, 3.43; increased insurer trust regression)” Balkrishnan (2004, p.818) “Social trust in physicians (beta =.329, t = 10.2, R2 =.443, p =.000) and satisfaction with the health-care insurer (beta =.491, t = 15.4, p =.000, R2 =.122) were strong predictors.” Gabay (2015, p. 87)	[19,23]
	Provider’s perceived quality	Positive	“These insurance problems could be primarily caused by the health care provider offering poor service” Fenenga (2015, p. 8)	[18,26]
Outcomes – reflecting behaviours	Choice: product choice/ acceptance of selective contracting	Positive	“Our findings indicate that reputation is fairly important in product choice when compared with product-based attributes” Kick *et al.* (2015, p.11) “The more trust enrolees have in their health insurer, the more accepting they are of selective contracting by their health insurer. The effect of specific trust is stronger compared to general trust.” Bes *et al.* (2013, p. 6)	[24,39,40]
		No association	“The results show that enrolees’ trust, both in general, and specifically in their health insurer and its purchasing strategy, is not associated with their choice for a policy with restrictive conditions.” Van der Hulst (2023, p.10)	[41]
	Policyholder portfolio: enrolment/change insurance plan/retention/drop-out	Positive (negative for drop-out)	“Likewise, not trusting the CBHI committee (governing bodies) increases the dropout rate from CBHI among rural HHS becoming ten times more likely compared to HHS who trust” Zepre *et al.* (2022, p. 9)	[18,42,24,26,29,37,38,43–53,54]
Outcomes – reflecting attitude	Willingness to pay	Positive	“Trust in the health insurance system is significantly associated with the amount stated by the respondents, i.e., the higher the trust the higher the amount willing to pay for health insurance” Myint (2019, p. 354)	[55,56]
	Data sharing	Positive	“trust positively influences willingness to share (b = 0.45, p < 0.01)” Seiferth (2020, p. 3717)	[21,35]
	Willingness to approach health insurer for healthcare advice	Positive	“Enrollees with more trust in the health insurer were more willing to approach their health insurer for healthcare advice” Van der Hulst (2023, p. 7)	[9]
	Attitude: active membership/ attitude/ seeking care from a non-PCP physician/ satisfaction	Positive	“Respondents with a higher level of insurer trust at baseline were less likely to use a non-PCP” Balkrishnan *et al.* (2004, p. 818)	[18,23,51,57–59]

#### 3.2.1. Determinants.

A total of 20 studies have explored the factors that influence the reputation of health insurance providers, revealing a diverse range of determinants. To provide a comprehensive understanding of the findings, the determinants were grouped into three distinct categories based on their relevance to specific stakeholder groups. First, policyholders rely on factors such as demographics, health literacy, and satisfaction levels, which directly influence their trust and perception of insurers. Second, health insurers include organizational factors under the control of insurers, including service quality, communication strategies, transparency, and corporate culture. Third, healthcare providers encompass variables related to the quality of care, trust in providers, and their communication styles, which indirectly affect the reputation of insurers.

3.2.1.1. *Determinants related to policyholders:* Six surveys focused on the characteristics of policyholders, such as age, gender, family status, income level, religious affiliation, and ethnicity, and their relationship with trust in health insurers [19–24]. They were conducted between 2003 and 2020 found no or minor association with reputation or trust. Bes [24] reported no significant association between gender and trust. Similarly, Goold [20] found no connection between age, race, income level, and trust in insurers, highlighting a discrepancy with most studies where race has been associated with trust in doctors, medical research, and the broader healthcare system (21 pp. 69, 71). These may partly reflect contextual variations across countries, where socio-demographic factors such as gender or ethnicity interact differently with individual and institutional trust.

One demographic factor, education or health literacy, consistently appeared to play a role in shaping trust. This conclusion was supported by six surveys [19,23,26,27,29,30], two qualitative interview [25,28], and one mixed-method study [18] conducted between 2006 and 2025 across divers countries. Eseta (26 p. 9) found that higher education levels correlated with increased trust, a finding echoed by Mathur (27 p. 10), who observed higher trust among those with greater health insurance literacy. However, Goold’s 2006 survey (21 p. 69) in the USA presented a contrasting view, suggesting that individuals with lower education levels might have higher trust, although she also noted that greater knowledge of the insurance plan enhanced trust. Such discrepancy might be explained by different understandings of trust across educational groups, or to country-specific levels of insurance literacy and exposure to the health system.

The same surveys that studied demographic variables have investigated the association between health status and trust. According to Gabay (20 p. 87), health status has a minimal influence on trust in the insurer. Goold (21 p. 69) states that individuals who reported fair or poor health status exhibited lower levels of trust in their insurer compared to those who reported good, very good, or exceptional health status. These findings were consistent across studies conducted in Germany in 2020 [21] and the USA in 2003 [22]. However, Bes (25 p. 6) found no association between trust in health insurers and health status. This divergence may be due to differences in study populations, sample sizes, or contextual factors such as variations in healthcare system performance and insurance coverage across settings.

3.2.1.2. *Determinants related to insurers:* Some of the variables observed in the selected studies demonstrate a direct dependence with insurers. Policyholders’ satisfaction with their insurer, the quality of the services offered by the latter and the communication adopted all contribute to the health insurers’ reputation.

The association of satisfaction and reputation or trust is supported by five surveys conducted between 2002 and 2022 across different countries and continents. Eseta conducted a qualitative interview in Ethiopia in 2022 [25]. Each of these studies reported a positive association with reputation. This convergence of findings may indicate that satisfaction plays a meaningful role in shaping insurer reputation, despite differences in health system design and study settings. Similar patterns of convergence were observed for several other insurer-related determinants described below.

Quality or perceived service quality were examined in four of the aforementioned studies [19,25,31,32], as well as in eight other surveys conducted between 2002 and 2022 in five countries and four continents [34,18,24,26,33,35,36,55], along with one mixed-method study carried out in Cambodia [38]. All of these studies concluded a positive association between quality and reputation. Mathur (2018) also observed a positive association between convenience and cost of plan (27 p. 7). Choice in product or plan is another variable positively associate with trust. One survey conducted in USA and a study using a mixed-method approach done in Ghana reach consistent results [18,22].

Communication has been consistently associated to reputation and trust. A positive association is supported by five surveys conducted between 2002 and 2022, across divers health systems [21,24,31,33,35], along with a qualitative study in Ghana (2015) and a mixed-method study in Cambodia (2009) [18,38]. Findings from Seiferth indicate that company publicity increases trust (22 p. 3716), while Grundstrom highlights the influence of showing empathy and a transparent communication in fostering trust (35 p. 8). The methodological diversity of these studies may strengthen the robustness of this association.

Eseta [25] examined drop-out from the community-based health insurance and observed a negative association with trust. No significant association was found between trust and selection of the health insurer, as examined in studies by Gabay [19] and Eseta, or between trust and locus of control, as investigated by Gabay.

Trust is be considered as a component of reputation by Mathur, who examines in a survey [26] the variance of seven broad factors, which includes 24 statements, explaining the model studied. In this study, along with determinants such as plan cost and quality of service, trust is related to perceptions and beliefs. According to Mathur, trust accounted for 7% of the variability in reputation and measured the level of “faith” in plans and insurance providers (27 p. 6). This may reflect both the distinction previously discussed between trust and reputation and the heterogeneity of definitions used for these concepts across studies.

3.2.1.3. *Determinants related to providers:* Variables linked to providers were also examined in the selected studies. They are trust in physicians, perceived quality of providers and their communication. Three surveys done in Israel, India and USA [19,23,26] as well as one study using a mixed-method done in Ethiopia [18] observed a positive association between these variables and reputation, except for the providers’ communication, where no association was found. Gabay did not observed a significant association between perceived communication and trust [19].

Trust in the primary care physicians also appears to be a key factor associated with trust in the health insurers according to Gabay (20 p. 87). Balkrishnan further supports that changes in primary care physicians are positively associated with trust in insurance companies (23 p. 818). In his study, Fenenga suggests, that a poor quality of health care providers can lower the trust in insurer [18]. Taken together, these findings could potentially suggest that in systems where insurers and providers are closely linked, such as in integrated systems, provider performance may indirectly shape public perception of insurers.

Overall, ten determinants were identified as showing a consistent positive association with reputation (such as satisfaction, cost of plan, choice and communication), while one was linked to a possible negative association. Four determinants, including demographic variables (such as age and gender), locus of control, selection or type of health insurance, and providers’ communication style, showed no significant association with reputation. Two determinants present conflicting results. The association between education and literacy appears to be either negative or positive, depending on the study. Study results also vary for health status, showing either no association or a positive association.

#### 3.2.2. Outcomes.

The majority of the studies, 32 in total, focus exclusively on or include an analysis of the outcomes related to insurer’s reputation. These studies have been categorized into seven sub-groups: choice (product and selective contracting); policyholder portfolio (enrolment, change insurance plan, retention and drop-out); willingness to pay; data sharing; willingness to approach health insurer for healthcare advice; attitude (active membership, seeking care from a non-primary care physician). To facilitate a comprehensive analysis of the results, the identified outcomes were classified into two distinct groups: those pertaining to behaviour and those associated with attitude.

The positive association between reputation and product choice or acceptance of selective contracting is supported by three studies [24,39,40]. According to Bes, greater trust among policyholders in their health insurance provider leads to increased acceptance of selective contracting by the insurer [24 p. 6]. Van der Hulst obtained contradictory results. He found no association between trust (in the industry or a specific insurer) and the choice of a policy with selective contracting [41 p. 10]. As both studies were conducted in Netherlands, the contradictory findings cannot be readily attributed to systemic or cultural differences. Moreover, differences in analytical focus are unlikely to explain this divergence, as both studies examine specific insurers rather than the insurance system as a whole. Finally, both studies focus on reputation rather than trust, further limiting the plausibility of conceptual differences as an explanation.

The most frequently studied outcome relates to the policyholder portfolio, encompassing enrolment, changes in insurance plans, retention, and drop-out, and was examined in 19 studies [18,42,24,26,29,37,38,43–53,54]. Across 11 countries representing diverse geographical and institutional contexts (S2 Appendix), these studies consistently observed positive associations with enrolment, plan changes, and retention, and a negative association with drop-out. This convergence of findings suggests that insurer reputation plays a meaningful role in shaping policyholder behaviour, beyond specific health system configurations.

Attitude, such as active membership and seeking care from non-primary care physicians, were examined in six studies carried out between 2004 and 2025, across 5 countries and continents [18,23,51,57–59]. Balkrishnan’s research suggests that higher levels of trust in an insurer increase the likelihood of seeking care from specialists rather than relying primarily on general practitioners (24 S. 818). In contrast to six studies reporting a positive association in which satisfaction is identified as a determinant of trust, Bhojak described an inverse directional relationship, with trust emerging as a determinant of satisfaction (57 S. 639).

One study, conducted in Myanmar in 2019 by Myint [55], and one in Tanzania by Nzowa [56], focused on willingness to pay and found a positive association with trust. According to Myint, the level of trust in the health insurance system is strongly correlated with the willingness to pay. The findings indicate that the higher the trust in the health insurance system, the greater the amount individuals are willing to pay for coverage (53 S. 354).

Finally, recent surveys explored the propensity to share data and seek healthcare advice from insurers [9,21,35]. All of these studies observed a positive relationship between these behaviors and insurer reputation.

## 4. Discussion

This scoping review offers a comprehensive overview of determinants and outcomes of insurer reputation. By clarifying the determinants that shape an insurer’s reputation and assessing their potential relevance, this study provides valuable insights for insurers seeking to enhance their reputational standing and for policymakers aiming to promote improved health outcomes. Finally, the research identifies existing gaps in the literature, thereby offering a foundation for future inquiries to contribute to a more comprehensive understanding of health insurers’ reputation. Detailed characteristics and associations for each determinant and outcome are presented in Table 1 and the Results section; the discussion below focuses on their interpretation, contextualization, and broader implications.

A first observation relates to the methodological approaches adopted, with a predominance of quantitative surveys over qualitative or mixed-methods designs. While such approaches offer scalability and statistical representativeness, their predominance is somewhat unexpected given the complexity and context-dependent nature of concepts such as reputation and trust.

A second observation concerns inconsistencies in the associations observed. Among the determinants, health literacy and health status warrant further consideration. One of the outcomes, choice (product choice, acceptance of selective contracting), is also subject to debate. In addition, inconsistencies emerge with regard to the directional relationship between certain closely related constructs, particularly trust and satisfaction. While most studies report a positive association in which satisfaction is identified as a determinant of trust, one study suggests an inverse relationship, with trust emerging as a determinant of satisfaction. This divergence points to an important conceptual and methodological challenge in the literature. Trust and satisfaction are closely intertwined constructs, and their temporal ordering is not always clearly specified. Depending on study design, analytical approach, and underlying theoretical assumptions, trust may be conceptualized either as an outcome of service experiences or as a pre-existing attitudinal disposition shaping subsequent satisfaction evaluations. The predominance of cross-sectional survey designs further limits the ability to disentangle these causal pathways.

These variations may stem from methodological heterogeneity across studies, including differences in sampling strategies, data collection modes, and analytical approaches. They may also reflect contextual influences, such as variations in health system structure, financing models, and cultural expectations regarding the roles of insurers and healthcare providers (S3 Appendix). Furthermore, conceptual definitions of trust and reputation are not always consistent across studies, which may lead to differences in measurement and interpretation. Acknowledging these potential sources of variation is important for situating the findings within their specific contexts and for informing the design of future research aiming to examine these determinants in a more comparable manner.

A third key finding involves mapping the determinants that shapes the reputation of health insurers. Indeed, while existing literature includes reviews on the concept of reputation, to the best of our knowledge, this study represents the first review specifically focused on the reputation of health insurers, providing an in-depth examination of the reputation of health insurance. Five reviews addressing health insurance and including the concept of reputation have been identified; however, reputation was not the central focus of these studies. Three studies addressed the barriers and facilitators associated with obtaining or renewing insurance [60–62]. Miti investigates the factors associated to individuals’ willingness to pay [63] and Conde analyses the factors that may influence the adoption of community health insurance [64]. As a result, several aspects related to the reputation of health insurers may have been overlooked.

Ten determinants emerged as consistently and positively associated with insurer reputation, most of which relate directly to insurers’ operations (e.g., corporate culture, service quality, communication, product structure). Knowing these determinants is crucial for health insurers’ managers, because they have a broad scope for action on these variables. The detailed breakdown of these associations is provided in Table 1; here, we focus on their strategic implications for insurers.

Health insurers have limited, or primarily indirect, influence over determinants related to policyholders and healthcare providers (e.g., providers’ perceived quality, communication, education, and health status). Despite limited control over these reputational determinants, understanding which factors that may contribute to reputation is crucial. Health insurers should consider initiatives to enhance their clients’ health literacy [65] and recognize the potential reputational relevance of healthcare providers’ quality [66]. This is particularly relevant in countries where the contractual relationship between providers and insurers is critical. Studies in the United States and Israel have explored these issues, and similar trends are emerging in other countries as capitation model projects gain prominence. Switzerland, though likely in an early stage, has initiated several projects aligned with this model (for example in Arc jurassien [67] or in Canton de Vaud [68].

A fourth key finding addresses the question of why reputation should be considered an important factor by both insurers and policymakers. First, the analysis of the outcomes reveals that the reputation of health insurers has a significant influence on various aspects of consumer behaviour, including dropout rates, retention, and enrolment. It is also associated with product selection, pricing and willingness to pay. These findings are consistent with broader research in insurance markets, which conceptualizes trust as an intangible asset shaping policyholder behaviour beyond purely contractual or financial considerations [69]. Considering the potential impact of reputation on these elements, it seems essential for health insurance managers to include their reputation in their development strategy [1,12]. Second, the willingness to receive health advice and the willingness to share data are also positively associate with trust. It should alert policymakers in terms of public health [70]. Finally, the analysis of reputation outcomes highlights another point through its gaps. None of the identified studies address the consequences in terms of policy. That could suggest a potential gap in the literature, concerning for instance the regulatory implications of insurers’ reputational standing. This issue is particularly important when considering its broader implications for public health. It could be hypothesized that a poor reputation among health insurers may lead to increased regulatory scrutiny, which could, in turn, affect the effectiveness of collaborative efforts between health insurers and other stakeholders within the healthcare system. Recent examples highlight this, especially in the realm of data processing – a subject briefly addressed in the reviewed studies, but primarily from the perspectives of policyholders and regulatory authorities. For instance, in 2024 and 2025, the Swiss parliament will deliberate on this issue, as evidenced in the referenced article [71].

Our findings resonate with broader debates in health services research and organizational behavior. Reputation, as explored in our study, may be understood not only as an outcome of service quality and communication but also as a potential strategic asset in health system governance. Theories of organizational behavior highlight how reputation contributes to institutional legitimacy [72], stakeholder trust [73,74], and competitive positioning [75]. In this light, health insurers’ efforts to cultivate a positive reputation align with broader principles of organizational identity and strategic management. Moreover, the observed emphasis on empathy, transparency, and service responsiveness links directly with patient-centered care models in health services research [76]. By bridging the fields of organizational studies and health services, our scoping review may provide insights into how insurer reputation might function both as a determinant and as a lever of system-level performance.

## 5. Implications

The findings of this review underscore significant implications for insurers, policymakers, and researchers, shaping a broader narrative around the importance of reputation in health insurance. For insurers, the evidence points to a clear mandate: understanding and addressing the determinants of reputation is not just an operational necessity but a strategic imperative. Insurers hold considerable control over several key determinants, including corporate culture, service quality, and product convenience. By fostering empathy and transparency within their organizations, enhancing the tangible benefits of their insurance products, and ensuring consistent, high-quality service delivery, insurers can directly influence their reputational standing. Moreover, strategic communication emerges as a pivotal tool. Clear, empathetic, and transparent messaging not only strengthens trust but also positions insurers as proactive partners in the healthcare journey of their clients.

Policymakers, too, have a vital role to play in recognizing the intersection of health insurer reputation with public health objectives. The findings suggest that reputation is not merely a business concern but a public health asset, influencing behaviours such as data sharing, health advice-seeking, and preventive care. For instance, in contexts like Switzerland, where debates around health insurer advertising and data privacy are ongoing, there is an opportunity to align regulatory frameworks with strategies that enhance reputational trust. Policymakers could also encourage initiatives that improve client health literacy or incentivize quality improvements among healthcare providers, recognizing that these efforts contribute not only to public health but also to the reputational resilience of insurers.

For researchers, this review highlights a critical gap in the existing body of knowledge. The predominance of survey-based studies underscores the need for more diverse research methodologies, particularly qualitative approaches that can capture the nuanced perspectives of policyholders, providers, and insurers. Future research should also explore the interplay between reputation and regulatory frameworks, shedding light on how trust in insurers influences policy outcomes and public health effectiveness. By adopting a more holistic and interdisciplinary approach, researchers can help build a comprehensive understanding of the mechanisms that underpin health insurer reputation, its determinants, and its far-reaching implications.

In sum, this narrative emphasizes the interconnected roles of insurers, policymakers, and researchers in leveraging reputation as a pivotal factor in shaping the future of health insurance and public health systems. Through coordinated efforts, these stakeholders can foster a more trusted and effective healthcare ecosystem.

## 6. Limitations

Specific limitations should be considered when analysing and interpreting the findings of this scoping review. This study aims to present an extensive overview of the existing literature regarding the broad topic of health insurance reputation. We are aware that the structure of a country’s health system affects how the subject is approached, leading to variation across studies. Significant variability is also observed in the methodologies of the studies, as well as in the number of individuals surveyed or interviewed, respectively. This limits any direct comparison. Additionally, the quality of identified studies varies, and the review does not include a critical appraisal, which may have resulted in the inclusion of lower-quality data. This factor should also be considered while interpreting the data. Another limitation is the inherent risk of missing relevant research. Despite employing carefully selected terms to capture the breadth of literature on this topic, the potential for overlooking some studies cannot be entirely eliminated. By including grey literature in our exclusion criteria, we may have introduced a publication bias, which could have resulted in an incomplete mapping of the available evidence. This should be taken in consideration, as the exclusion criteria of absence of “insur*”, “health plan” or “managed care” in the title. Manual screening was carefully conducted to minimize the risk of missing relevant records from related fields such as communication, health policy or health sciences; however, this risk cannot be entirely ruled out. As a result, certain strands of the literature may be underrepresented, which could have influenced the comprehensiveness and thematic structure of the evidence identified. This review relied on three databases, and while these were selected for their relevance and coverage, expanding the number of databases could have enhanced the comprehensiveness of the review and potentially identified additional relevant studies. In addition, consistent with the objectives of a scoping review, we did not conduct a formal critical appraisal of the methodological quality of the included studies. As a result, studies of varying design and rigor were mapped without differential weighting, which limits conclusions regarding the relative strength of the evidence and the robustness of individual associations. Findings should therefore be interpreted with caution, particularly when informing policy or strategic decision-making. Future research could benefit from expanding the number of databases used and from conducting systematic reviews focused on specific determinants or outcomes, including a formal evaluation of study quality.

## 7. Conclusion

The current literature on health insurance reputation highlights significant gaps in comprehensive evaluations of its determinants and outcomes. This review identifies key factors influencing reputation – such as corporate culture, product structure, service quality, and provider quality – many of which insurers can directly control. By fostering transparency, empathy, and effective communication, insurers can positively shape their reputation and enhance policyholder trust.

A strong reputation not only supports core business operations, such as policyholder acquisition and retention, but also plays a critical role in promoting preventive care. However, existing studies predominantly focus on business outcomes, with limited attention to the regulatory implications of insurer reputation. Exploring these implications could provide valuable insights into how reputation may influence healthcare systems and public health.

Future research should address these gaps by examining the regulatory and public health consequences of insurer reputation. A deeper understanding in this area would help align insurer practices with broader healthcare strategies, improving both service delivery and the adoption of preventive health measures.

## Supporting information

S1 Appendix Database search blocks. (DOCX)

S2 Appendix Characteristics of the included studies. (DOCX)

S3 Appendix Health expenditure, % Current health expenditure (CHE) 2021. (DOCX)
