# Supplementary material for: Reputation and trust in health insurance: A scoping review of key drivers and outcomes
Source: PLoS One. 2026 Mar 27;21(3):e0345875. doi: 10.1371/journal.pone.0345875 (PMC13028422; doi:10.1371/journal.pone.0345875)
Supplement: S1 Appendix — (DOCX) [file pone.0345875.s001.docx]

**S1. Appendix. Database search blocks**

**PubMed**

((insur*[Title] OR "health plan*"[Title] OR "managed care"[Title]) AND (reputation[Title/Abstract] OR "corporate image"[Title/Abstract] OR "corporate identity"[Title/Abstract] OR "reputational status"[Title/Abstract] OR appeal[Title/Abstract] OR ethos[Title/Abstract] OR fame[Title/Abstract] OR esteem[Title/Abstract] OR "perceived quality"[Title/Abstract] OR prominence[Title/Abstract] OR trust[Title/Abstract]))

**Scopus**

( TITLE ( insur* OR "health plan*" OR "managed care" ) AND TITLE-ABS-KEY ( reputation OR "corporate image" OR "corporate identity" OR "reputational status" OR appeal OR ethos OR fame OR esteem OR "perceived quality" OR prominence OR trust ) ) AND PUBYEAR > 1989 AND PUBYEAR < 2027

**Web of Science**

TI=(insur* OR "health plan*" OR "managed care") AND TS=(reputation OR "corporate image" OR "corporate identity" OR "reputational status" OR appeal OR ethos OR fame OR esteem OR "perceived quality" OR prominence OR trust)
