# Supplementary material for: Reputation and trust in health insurance: A scoping review of key drivers and outcomes
Source: PLoS One. 2026 Mar 27;21(3):e0345875. doi: 10.1371/journal.pone.0345875 (PMC13028422; doi:10.1371/journal.pone.0345875)
Supplement: S2 Appendix — (DOCX) [file pone.0345875.s002.docx]

**S2. Appendix. Characteristics of the included studies**

| **Frist author, year Country/countries of data collection (World Bank classification), Continent** | **Title** | **Mainly used concept** | **Methodology (tool)** | **Participants (responses)** | **Study object** | **Industry or insurers/government** |
| --- | --- | --- | --- | --- | --- | --- |
| R. E. Bes, 2013 (25) Netherland (high-income) , Europe | Acceptance of selective contracting: The role of trust in the health insurer | Trust | Survey (e-mail and per post) | Policyholders (n=4422) | Outcomes | Industry, insurers/government |
| G. Gabay, 2015 (20) Israel (high-income), Asia | Antecedents of Patient Trust in Health-Care Insurers | Trust | Survey (e-mail) | Policyholders (n=815) | Determinants | Insurers/government |
| N. T. Shiferaw, 2025 (49), Ethiopia (low-income), Africa | Assessing the impact of  community-based health insurance  on health service utilization and  out-of-pocket payments in Dangila  Wereda, Awi zone, Ethiopia | Trust | Survey (not specified) | Households (n=419) | Outcomes | Insurers/government |
| R. Banerjee, 2025 (50), India, Asia | Assessing What Matters Most in Health Insurance Choices: An  IPMA Study of Customer Attitude and Perceived Credibility  As Mediators | Trust | Survey (online and offline questionnaire) | Policyholders (n=421) | Outcomes | Insurers/government |
| G. Demissie, 2021 (51), Ethiopia (low-income), Africa | Barriers and Facilitators of Community-Based Health Insurance Membership in Rural Amhara Region, Northwest Ethiopia: A Qualitative Study | Trust | Qualitative (focus group) | Farmers and key informants (n=70) | Outcomes | Insurers/government |
| O. D. Kibu, 2024 (48) Cameroon (Lower-middle-income), Africa | Barriers and Motivations for Health Insurance Subscription Among Health-Care Users in Cameroon | Trust | Qualitative (focus group and interview) | Healthcare users (n=37) | Outcomes | Insurers/government |
| S. Ozawa, 2018 (29), Cambodia (lower-middle-income), Asia | Building informed trust: developing an  educational tool for injection practices and  health insurance in Cambodia | Trust | Qualitative: workshops and face to face interview | Villagers (n=480) | Determinants | Insurers/government |
| W. Cheng, 2022 (32) China (upper-middle-income), Asia | Construction and validation of a revised satisfaction index model for the Chinese urban and rural resident-based basic medical insurance scheme | Trust, satisfaction, perceived quality | Survey (not specified) | Students (n=1909) | Determinants, assessment | Insurers/government |
| S. Wendel, 2011 (16) Netherland, (high-income), Europe | Consumer evaluation of complaint handling in the Dutch health insurance market | Trust, satisfaction | Survey (per post) | Policyholders (n=150) | Determinants | Insurers/government |
| T. Sadigov, 2024 (52), Azerbaijan (upper middle-income), Europa | Cost of a Policy: Social Trust and Health Insurance. Uptake in Azerbaijan and Emerging Markets | Trust | Mixed method: quantitative (multivariable OLS regression) and qualitative (focus group) | Policyholders and non-policyholders (n=24) | Outcomes | Insurers/government |
| L. Tam, 2021 (55) Australia (high-income), Oceania | Determinants of attitude and intention towards private health insurance: a comparison of insured and uninsured young adults in Australia | Trust | Survey, (online questionnaire) | Young-adults (n=583) | Outcomes | Industry |
| E. Nshakira-Rukundo (58), 2019 Uganda (low-income), Africa | Determinants of enrolment and renewing of community-based health insurance in households with under-5 children in rural South-Western Uganda | Trust, perception | Survey (online questionnaire) | Policyholders, household (n=464) | Outcomes | Insurers/government |
| H. O. Oriakhi, 2012 (43) Nigeria(lower-middle-income), Africa | Determinants of rural Household's willingness to participate in community-based health insurance scheme in Edo state, Nigeria | Trust | Survey (trained enumerators) | Households (n=360) | Outcomes | Industry |
| B. Zheng, 2002 (34) USA (high-income), North America | Development of a scale to measure patients' trust in health insurers | Trust | Survey (telephone) | Policyholders (n=410, n=1152) | Determinants, assessment | Insurers/government |
| E. Dugan, 2005 (76) USA (high-income), North America | Development of abbreviated measures to assess patient trust in a physician, a health insurer, and the medical profession | Trust | Survey (telephone) | Policyholders (n=1117, n=1024) | Assessment | Insurers/government |
| M. Kick, 2015 (40) Germany (high-income), Europe | The effect of corporate reputation on health insurance choices in a public-policy-shaped environment of premium equality | Reputation | Survey, (online questionnaire) | Policyholders (n=250) | Outcomes | Insurers/government |
| E. Nsiah‐Boateng, 2024 (47) Ghana (lower-middle-income), Africa | Effect of social capital on enrolment of informal sector occupational groups in the national health insurance scheme in Ghana: a cross‐sectional survey | Trust | Survey (questionnaire, not specified) | Informal sector occupational groups (n=528) | Outcomes | Insurers/government |
| M. Kick 2015 (39) Germany (high-income), Europe | The effects of additional contributions on statutory health insurance choices in Germany | Reputation | Survey (online questionnaire) | Policyholders (n=250) | Outcomes | Insurers/government |
| T. Mathur, 2018 (27) India (lower-middle-income), Asia | Examining the influence of health insurance literacy and perception on the people preference to purchase private voluntary health insurance | Perception, trust | Survey (questionnaire, not specified) | Households (n=300) | Determinants, outcomes, assessment | Insurers/government |
| W. A. Eseta, 2022 (26) Ethiopia (low-income), Africa | Factors affecting households' trust in the community-based health insurance scheme in Ethiopia | Trust, perceived quality, satisfaction | Survey (face-to-face questionnaire) | Policyholders, household (n=634) | Determinants | Insurers/government |
| K. Zepre, 2022 (46) Ethiopia (low-income), Africa | Factors influencing drop-out of households from community-based health insurance membership in rural districts of Gurage Zone, Southern Ethiopia: Community based case-control study | Trust | Mixed method: Survey (interviewer-administered), focus group discussions | Households (n=525, n=6) | Outcomes | Insurers/government |
| H. M. A. Khuwaja, 2021 (18) Pakistan (lower-middle-income), Asia | Factors influencing low enrollment in a community-based health insurance scheme, Karachi, Pakistan: A mixed methods case study | Trust | Mixed method: Survey (investigator derived), qualitative interviews | Policyholders and non-policyholders (n=10, n=17, n=190) | Outcomes | Industry |
| K. Jayaraman, 2017 (42) Malaysia (upper-middle-income), Asia | Factors influencing the purchase intention of health insurance policy-an empirical study in Malaysia | Reputation, perception | Survey (filled–in questionnaires) | Policyholders (n=105) | Outcomes | Insurers/government |
| E. L. Okiche, 2021 (19) Nigeria (lower-middle-income), Africa | Health care payment practice, perception and awareness of national health insurance scheme by market women in Enugu Metropolis South-East Nigeria | Trust, perception | Qualitative: semi-structured interviewer questionnaire | Women traders who were aged 18 years and above (n=353) | Assessment | Insurers/government |
| C. Y. Myint, 2019 (53) Myanmar (lower-middle-income), Asia | Health insurance in Myanmar: Knowledge, perceptions, and preferences of Social Security Scheme members and general adult population | Trust | Survey (face to face) | General population and policyholders (n=640) | Outcomes | Insurers/government |
| P. Kautish, 2022 (37) India (lower-middle-income), Asia | Health insurance policy renewal: an exploration of reputation, performance, and affect to understand customer inertia | Reputation | Structured survey (online) | Policyholders (n=228) | Determinants, outcomes | Insurers/government |
| J. P. Frank, 2023 (41) Netherland, (high-income), Europe | How is enrollees’ trust in health insurers associated with choosing health insurance? | Trust | Survey (online or by post questionnaire) | Policyholders (n=1125) | Outcomes | Industry/Insurers/government |
| S. Kadyan, 2022 (36) India (lower-middle-income), Asia | Impact of claim settlement procedure of health insurance companies on customer satisfaction during the pandemic: A case of third-party administrators | Satisfaction, trust, (reputation) | Survey (e-mail per post, curried) | Policyholders (n=430) | Determinants | Insurers/government |
| A. Singh, 2025 (30), India (lower-middle-income), Asia | Impact of health insurance literacy, brand reputation, and risk attitude on  intentions to purchase private health insurance policy | Reputation, trust, satisfaction | Survey (online) | Residents (n=229) | Determinants, outcomes | Insurers/government |
| N. Bhojak, 2023 (57), India (lower-middle-income), Asia | Impact of trust, sales agent and service delivery on health insurance holder satisfaction and experience | Trust | Survey (not specified) | Policyholders (n=418) | Outcomes | Insurers/government |
| V. F. Raza, 2017 (44) Pakistan (lower-middle-income), Asia | Impressions and attitudes of adult residents of Karachi towards a possible public health insurance scheme | Trust | Survey (questionnaire, not specified) | Permanent residents (n=340) | Outcomes | Insurers/government |
| C. Grundstrom, 2020 (35) Finland (high-income), Europe | Insurance customers' expectations for sharing health data: Qualitative survey study | Trust | Survey (online) | Policyholders (n=452) | Determinants | Insurers/government |
| U. Sebjan, 2013 (45) Slovenia (high-income), Europe | Key factors in the decision-making process for complementary voluntary health insurance | Reputation | Survey (visited) | Policyholders (n=300) | Outcomes | Insurers/government |
| S. D. Goold, 2006 (21) USA (high-income), North America | A measure of trust in insurers | Trust | Structured survey (telephone) | Policyholders (n=4005) | Determinants, outcomes, assessment | Industry |
| P. G. Nzowa, 2023 (54) Tanzania (low-income country), Africa | Mediation effect of trust on willingness  to pay for health insurance among co‑operative  members in Tanzania | Trust | Survey (not specified) | Policyholders (n=497) | Outcomes | Insurers/government |
| P. Perrow (2023) (56) South-Africa, (uper-middle-income), Africa | Mistrust in government and National Health Insurance: A qualitative study of solo private practitioners in Cape Town | Trust | Qualitative: semi-structured interviewer questionnaire | General practitioners (n=9) | Outcomes | Insurers/government |
| X. Liu, 2020 (33) China (upper-middle-income), Asia | Mixed methods research on satisfaction with basic medical insurance for urban and rural residents in China | Trust, satisfaction | Survey (telephone) | Policyholders (n=1335, n=23) | Determinants | Insurers/government |
| R. Peng, 2022 (28) China (upper-middle-income), Asia | Public trust in the long-term care insurance pilot program in China: An analysis of mediating effects | Trust | Survey (online) | Residents (n=786) | Determinants | Industry |
| F. J. P. van der Hulst, 2023 (9) Netherland (high-income), Europe | The relation between trust and the willingness of enrollees to receive healthcare advice from their health insurer | Trust | Survey (online or by post) | Policyholders (n=885) | Outcomes | Insurers/government |
| van der Hulst F. J. P., 2024 (31), Netherland (high-income), Europe | The relationship between enrollees’ perceptions of health insurers’ tasks and their trust in them | Trust | Survey (online) | Policyholders (n=837) | Determinants | Insurers/government |
| A. Seiferth, 2020 (22) Germany (high-income), Europe | Sharing personal health and fitness data with health insurance providers: An empirical study considering trust and risk | Trust | Survey (online), between-subject experiment | Policyholders (n=238) | Determinants, outcomes | Insurers/government |
| C. J. Fenenga, 2015 (17) Ghana (lower-middle-income), Africa | Social capital and active membership in the Ghana National Health Insurance Scheme - A mixed method study | Trust, social capital | Mixed method: Interviews, focus group discussions, household survey (not specified) | Policyholders & non-policyholders (Interview n=20), focus group (n=22), Key informants (n=7), survey (n=7097) | Determinants, outcomes | Insurers/government |
| R. Balkrishnan, 2004 (24) USA (high-income), North America | Trust in insurers and access to physicians: Associated enrollee behaviors and changes over time | Trust | Survey (telephone) | Policyholders (n=1918, n=558) | Determinants, outcomes, assessment | Insurers/government |
| R. Balkrishnan, 2003 (23) USA (high-income), North Amerika | Trust and Satisfaction with Physicians, Insurers, and the Medical Profession | Trust | Survey (telephone) | Policyholders (n=1117) | Determinants | Insurers/government |
| S. Ozawa, 2009 (38) Cambodia (lower-middle-income), Asia | Trust in the context of community-based health insurance schemes in Cambodia: Villagers' trust in health insurers | Trust | Mixed method: focus groups and surveys (not specified) | Households (n=535) Focus Group (n=74) | Determinants, outcomes, assessment | Insurers/government |
