# Supplementary material for: Reputation and trust in health insurance: A scoping review of key drivers and outcomes
Source: PLoS One. 2026 Mar 27;21(3):e0345875. doi: 10.1371/journal.pone.0345875 (PMC13028422; doi:10.1371/journal.pone.0345875)
Supplement: S3 Appendix — (DOCX) [file pone.0345875.s003.docx]

**S3. Appendix. Health expenditure, % Current health expenditure (CHE) 2021**

Based on the hypothesis that the economic situation of a country and the way in which the healthcare system is financed can have an impact on the reputation of health insurance, it also seemed interesting to take a closer look at the countries in which the studies were carried out. Based on World Bank classification, of the 45 studies, the majority (15) were conducted in high-income countries, three in upper-middle-income countries, 12 in lower-middle-income countries, and three in low-income countries. While the distribution is unequal, the selected studies were conducted in very different context.

Data from the World Health Organization indicates that among the 19 countries represented, six rely primarily on compulsory insurance systems, seven operate government-based schemes, and six predominantly depend on out-of-pocket payments for healthcare (Appendix III). However, no definitive conclusion could be drawn from this analysis.

| **Country** | **Government schemes** | **Compulsory contributory health insurance schemes** | **Voluntary health care payment schemes** | **Household out-of-pocket payments (OOPS)** |
| --- | --- | --- | --- | --- |
| Australia | 73 | 1 | 12 | 14 |
| Azerbaijan | 32 | 0 | 0 | 68 |
| Cambodia | 36 | 2 | 7 | 55 |
| Cameroon | 23 | < | 10 | 67 |
| China | 17 | 37 | 11 | 34 |
| Ethiopia | 46 | 0 | 17 | 37 |
| Finland | 67 | 13 | 4 | 16 |
| Germany | 11 | 75 | 3 | 12 |
| Ghana | 49 | 11 | 13 | 27 |
| India | 34 | 2 | 14 | 50 |
| Israel | 19 | 49 | 11 | 20 |
| Malaysia | 56 | 1 | 12 | 32 |
| Myanmar | 19 | 1 | 10 | 70 |
| Netherland | 13 | 72 | 6 | 9 |
| Nigeria | 13 | 1 | 10 | 76 |
| Pakistan | 32 | 1 | 10 | 57 |
| Slovenia | 13 | 61 | 13 | 13 |
| South Africa | 50 | . | 45 | 6 |
| Tanzania | n/a | n/a | n/a | n/a |
| Uganda | 45 | 0 | 23 | 31 |
| US | 30 | 53 | 6 | 11 |

Source: World Health Organization. Global Health Expenditure Database: https://apps.who.int/nha/database/ViewData/Indicators/en
